# Ethanol-Related Behaviors in Mouse Lines Selectively Bred for Drinking to Intoxication

**DOI:** 10.3390/brainsci11020189

**Published:** 2021-02-04

**Authors:** Bryan E. Jensen, Kayla G. Townsley, Kolter B. Grigsby, Pamela Metten, Meher Chand, Miracle Uzoekwe, Alex Tran, Evan Firsick, Katherine LeBlanc, John C. Crabbe, Angela R. Ozburn

**Affiliations:** Department of Behavioral Neuroscience, Oregon Health & Science University, and VA Portland Health Care System, Portland, OR 97239, USA; jensebry@ohsu.edu (B.E.J.); kayla.townsley@icahn.mssm.edu (K.G.T.); grigsbyk@ohsu.edu (K.B.G.); mettenp@ohsu.edu (P.M.); meherchandbarwal@gmail.com (M.C.); miracle.uzoekwe@gmail.com (M.U.); alexandertdtran@gmail.com (A.T.); firsick@ohsu.edu (E.F.); kleblanc@stanford.edu (K.L.); crabbe@ohsu.edu (J.C.C.)

**Keywords:** mice, drinking in the dark, selected lines, operant self-administration, ethanol, sensitivity to ethanol

## Abstract

Alcohol use disorder (AUD) is a devastating psychiatric disorder that has significant wide-reaching effects on individuals and society. Selectively bred mouse lines are an effective means of exploring the genetic and neuronal mechanisms underlying AUD and such studies are translationally important for identifying treatment options. Here, we report on behavioral characterization of two replicate lines of mice that drink to intoxication, the High Drinking in the Dark (HDID)-1 and -2 mice, which have been selectively bred (20+ generations) for the primary phenotype of reaching high blood alcohol levels (BALs) during the drinking in the dark (DID) task, a binge-like drinking assay. Along with their genetically heterogenous progenitor line, Hs/Npt, we tested these mice on: DID and drinking in the light (DIL); temporal drinking patterns; ethanol sensitivity, through loss of righting reflex (LORR); and operant self-administration, including fixed ratio (FR1), fixed ratio 3:1 (FR3), extinction/reinstatement, and progressive ratio (PR). All mice consumed more ethanol during the dark than the light and both HDID lines consumed more ethanol than Hs/Npt during DIL and DID. In the dark, we found that the HDID lines achieved high blood alcohol levels early into a drinking session, suggesting that they exhibit front loading like drinking behavior in the absence of the chronicity usually required for such behavior. Surprisingly, HDID-1 (female and male) and HDID-2 (male) mice were more sensitive to the intoxicating effects of ethanol during the dark (as determined by LORR), while Hs/Npt (female and male) and HDID-2 (female) mice appeared less sensitive. We observed lower HDID-1 ethanol intake compared to either HDID-2 or Hs/Npt during operant ethanol self-administration. There were no genotype differences for either progressive ratio responding, or cue-induced ethanol reinstatement, though the latter is complicated by a lack of extinguished responding behavior. Taken together, these findings suggest that genes affecting one AUD-related behavior do not necessarily affect other AUD-related behaviors. Moreover, these findings highlight that alcohol-related behaviors can also differ between lines selectively bred for the same phenotype, and even between sexes within those same line.

## 1. Introduction

Alcohol use disorders (AUDs) pose a significant socioeconomic and public health problem worldwide. Alcohol misuse resulted in an estimated $249 billion in medical care expenses to the US in 2010. In the same year, the National Institute on Alcohol Abuse and Alcoholism (NIAAA) found that alcohol misuse was the number one factor in premature death and disability among 15- to 49-year-old US citizens (NIAAA; https://www.niaaa.nih.gov/publications/brochures-and-fact-sheets/alcohol-facts-and-statistics). A major contributor to the negative consequences of alcohol is attributed to binge drinking, which the NIAAA defines as a pattern of drinking that leads to a blood alcohol level (BAL) greater than 0.08 g/dL [1]. Binge drinking is a potent risk factor for the development of alcohol dependence and AUDs [2,3]. Considerable work has identified brain regions and molecular mechanisms underlying many aspects of AUD, including binge drinking [4,5,6]; however, AUD is a complex, polygenic disease and it is important to determine the generalizability of these findings across several species, strains, and drinking paradigms. The primary aim of these studies is to finely characterize and compare two replicate lines of mice that were selectively bred to drink to intoxication during a limited period of ethanol access (High Drinking in the Dark-1 and -2; HDID-1 and -2) and compare them to their genetically heterogenous founders, the Heterogeneous Stock/Northport (Hs/Npt) [7,8,9]. Traits on which both HDID lines differ from the non-selected Hs/Npt line can be presumed to reflect the influence of some of the same genes or gene networks underlying risk for DID. For example, these lines have been tested in a battery of tests [7,10,11,12,13]. Notably, HDID-1 and HDID-2 mice are less sensitive to the aversive effects of ethanol than Hs/Npt mice; however, no line differences were observed in sensitivity to the rewarding effects of ethanol [14].

Testing and modeling endophenotypes remains a critical tool in understanding the genetic basis of complex behaviors, such as binge-like ethanol drinking and AUDs [15]. Ethanol sensitivity, loss of control (binge drinking), tolerance, and relapse have been identified as important factors in evaluating endophenotypes throughout the stages of the AUD diagnosis [15]. Each stage of AUD progression is likely influenced by unique gene networks, which should be systematically considered in preclinical alcohol research [15]. The HDID lines represent genetic models of high risk for binge-like ethanol drinking. Therefore, an overarching goal of the current work is to determine whether the HDID lines differ from their Hs/Npt founders for other binge-like drinking endophenotypes. Specifically, we assessed (1) diurnal and temporal limited-access ethanol drinking, (2) sensitivity to the sedative effects of ethanol (using loss of righting reflex, LORR), (3) operant ethanol self-administration (under fixed ratio (FR) schedule of reinforcement), (4) ethanol seeking (using operant self-administration followed by extinction and cue-induced reinstatement procedures), and (5) the motivation for access to ethanol (using operant self-administration followed by a progressive ratio (PR) schedule of reinforcement).

Until the last decade, preclinical alcohol research in rodents has primarily involved voluntary consumption of ethanol in unlimited-access paradigms, where water is also an option [16]. In contrast to human binge-drinking behavior, rodents typically do not consume enough ethanol in these paradigms to reach pharmacologically meaningful BALs [7], but there are exceptions [17]. To model binge-like drinking, Rhodes et al. developed a limited-access paradigm [Drinking in the Dark (DID)], whereby C57BL6/J and other high-drinking strains reliably reach intoxicating BALs with 2–4 h of access to a single tube of 20% ethanol solution, offered 3 h into their dark cycle [18]. In comparing 12 inbred mouse strains, Rhodes et al. 2007 found that DID ethanol intake and BALs were strain dependent, suggesting a genetic contribution to binge-like drinking behavior. To study the genetic contribution to high binge-like ethanol drinking, the HDID-1 and HDID-2 lines were selectively bred to reach high BALs during the DID assay, during which both replicate lines reach average BALs of over 200% mg and show behavioral signs of intoxication [9,19].

C57BL6/J mice and other high-drinking strains have been shown to drink more ethanol in the dark phase than the light phase, which is one primary basis for assessing the DID assay three hours into the dark cycle [18,20]. While it is established that HDID-1 and HDID-2 drink more than their Hs/Npt founders during DID, it had not been determined whether the HDID lines maintain higher ethanol intake than Hs/Npt mice or will reach intoxicating BALs during the light. To determine whether selective pressure during the DID procedure altered drinking behaviors during all times of the day, we evaluated the diurnal drinking behaviors of HDID-1, HDID-2 and Hs/Npt by testing them in a DID and a drinking in the light (DIL) assay: following the same procedure as a DID but performing it at ZT3 (zeitgeber time 3; 3 h after lights on). This was designed to show us whether the genetic changes effected by enhancing the DID trait would alter drinking behaviors during all times of the day, or just during the animals’ most active and highly ingestive period, thus giving insight into their limited-access drinking behaviors.

To build on this, we also wanted to know when mice become intoxicated during a limited-access drinking assay. It has been previously shown that the HDID-1 and -2 lines are genetically distinct [21], and display markedly different temporal drinking patterns, where HDID-1 mice drink larger lick volumes (“gulpers”) while HDID-2 have higher lick rates (“sippers”) to achieve high BALs [22]. To better determine when these mice become intoxicated during the DID task, we tested separate cohorts of HDID-1, HDID-2, and Hs/Npt mice in a DID paradigm at 20 min intervals. By inference, this would tell us their patterns of drinking to intoxication. Following chronic bouts of repeated binge-like ethanol drinking using DID, C57BL/6J mice have been shown to exhibit “front-loading” behavior, in which greater volumes are consumed in the earlier phases of ethanol access [17,18,19], suggesting that they are drinking to achieve intoxication in a pattern that is excessive, and potentially harmful. If HDID lines reached 80 mg% more rapidly than Hs/Npt, this would allow us to conclude that the selected lines are “front loading” their ethanol intake, which would suggest that they exhibit patterns of drinking, previously only seen after chronic exposure.

Sensitivity to the intoxicating effects of ethanol, or the subjective response in humans, is a critical predictor of risk for AUD development, the genetic contributions to relative sensitivity have been widely tested through endophenotypes of ethanol-induced locomotor activity, ataxia, hypothermia, and LORR [15,23,24,25,26,27]. There exists a negative genetic correlation between LORR and voluntary ethanol consumption [28,29], i.e., some genes or gene networks predisposing low sensitivity also predispose high risk for AUD, and vice versa. It is not yet known whether such a relationship exists specifically for LORR and binge-like drinking. There is a steep dose–response curve for sensitivity to ethanol-induced LORR, and rather than testing multiple doses, three genotypes, and both sexes of mice to better understand how DID and LORR relate, sensitivity to ethanol was assessed using the “Up and Down Method” for determining the mean effective dose at which 50% of the population would lose their righting reflex (ED_50_) [30]. To test whether HDID selection altered sensitivity to the sedative effects of ethanol, ED_50_ for LORR was determined in the HDID-1, HDID-2, and Hs/Npt in the light and the dark (to allow for comparisons with limited-access drinking paradigms DIL, DID). If there is a negative genetic correlation for sensitivity to LORR and binge-like drinking (DID), both lines of HDID mice should exhibit a higher ED_50_ for LORR onset than Hs/Npt mice (specifically in the dark).

Operant ethanol self-administration in rodents is highly correlated with voluntary ethanol intake in many genetic animal models and can be used to explore ethanol seeking and motivational behaviors for ethanol—two critical and complex components of AUD [31]. Therefore, we determined whether HDID selection had any effects on ethanol self-administration behaviors compared to their Hs/Npt founders. To evaluate ethanol seeking, we tested whether these lines differed in cue-induced responding following a period of extinction training. In order to test the motivation, or willingness of these mice to “work” for access to 20% ethanol, we ascertained the highest response ratio reached in a PR session (defined as the “breakpoint”) among HDID-1, HDID-2 and Hs/Npt mice [32]. For these experiments, we would expect the HDID mice to exhibit higher levels of seeking behavior and motivation for ethanol access than Hs/Npt mice.

Taken together, this set of studies tested whether selection for high drinking in the dark behavior changed other traits or endophenotypes associated with increased risk for AUD. In comparing diurnal limited-access intake, we hypothesized that the HDID-1 and HDID-2 mice would show higher ethanol consumption compared to Hs/Npt during both light and dark phases, with the greatest intake for all lines occurring during DID. HDID mice show increased sensitivity to ethanol induced locomotor activity and ataxia [11], but many other reliable measures of the sensitivity to the intoxicating effects of ethanol remain to be tested. We hypothesized that both HDID lines will show a higher ED_50_ for LORR than Hs/Npt, suggesting a decreased sensitivity due to selection. Lastly, we hypothesized that compared to Hs/Npt, HDID-1 and HDID-2 mice will display higher ethanol seeking and higher motivation for ethanol access.

## 2. Materials and Methods

### 2.1. Animal Husbandry

Animals were bred and maintained in the VA Portland Health Care System Veterinary Medical Unit in standard polycarbonate cages (19 × 31 × 13 cm^3^) on Bed-o’cobs^®^ bedding (Andersons, Maumee, OH, USA) with stainless-steel wire bar tops with a recess for chow. Purina 5LOD chow (PMI Nutrition International, Brentwood, MO, USA) and water were available *ad libitum*, except when indicated, and cages were changed weekly. Animals were bred and maintained on a reverse 12 h light: 12 h dark cycle—lights on at 2030 (ZT0) and lights off at 0830 (ZT12)—and at a room temperature of 21 ± 1 °C. Prior to drinking studies, mice were group housed: 2–5 females or 2–4 males. Mice were aged 2–6 months old at time of study. Experimentally naïve mice were pseudo-randomized into experimental groups to ensure an even distribution of families among the groups for each experiment. All procedures were approved by the local Institutional Animal Care and Use Committee and were conducted in accordance with the NIH Guidelines for the Care and Use of Laboratory Animals.

### 2.2. Drugs

For the drinking studies, mice were offered 20% ethanol (*v*/*v*, in tap water; 200 proof, DeCon Labs, King of Prussia, PA, USA). For injections, 20% ethanol (*v*/*v*, in 0.9% saline) was administered interperitoneally (IP).

### 2.3. Drinking in the Dark (DID)/Drinking in the Light (DIL)

To test whether selection for BAL after binge-like drinking during lights off (active cycle for mice) generalized to increase intake during lights on, we measured limited-access drinking in the light (DIL) and dark (DID) in separate groups of male and female Hs/Npt, HDID-1, and HDID-2 mice. As in Rhodes et al., mice were habituated to individual housing and a new sipper bottle for one week prior to testing [18]. HDID-1 (S30.G32-S31.G33, where “S” is the number of selected generations, and “G” is the number of total generations), HDID-2 (S25), and Hs/Npt (G81.V13, where V is the number of generations of this non-selected, genetically segregating stock in our VA facility) mice were tested simultaneously in the same experimental room on the same reverse light cycle day (*n* = 8–9/sex/time of day/genotype). Mice were offered one bottle of 20% ethanol (*v*/*v* in tap water) for 2 h (from ZT3-5 for the DIL mice and ZT15-17 for DID mice) on days 1–3 and for 4 h (from ZT3-7 for DIL mice and from ZT15-19 for DID mice) on day 4. A 20 uL periorbital blood sample was collected immediately after ethanol access on day 4 and BALs were determined using gas chromatography (Agilent, Santa Clara, CA, USA) as previously described [33].

### 2.4. Relationship of DID and BAL over the 4 h DID Access Period

In order to assess temporal patterns of drinking to intoxication behaviors, we measured ethanol intake and BALs achieved throughout DID in separate cohorts of Hs/Npt, HDID-1, and HDID-2 mice in 20 min increments over the 4 h period on the final day of a 4 day DID test. 216 mice (*n* = 3/sex/genotype/time point) underwent a DID test for 4 days, as described above. On the final day, drinking amounts were recorded from separate groups of mice at 20 min intervals and blood was immediately sampled from their periorbital sinus (in a neighboring room), and compared with a final group sampled at the usual time of 4 h. Care was taken to leave the remaining animals undisturbed by quietly removing each time point group to a separate room just prior to rapid blood sampling. BALs were determined using gas chromatography, as described above.

### 2.5. ED_50_ Loss of Righting Reflex

To determine whether selection for drinking to intoxication during the dark altered diurnal sensitivity to ethanol, we determined the ED_50_ for onset of loss of righting reflex in Hs/Npt, HDID-1, and HDID-2 mice at ZT3 and ZT15. These testing times were selected because they correspond to the start of the DIL and DID times (above): 3 h after the lights on or off, respectively. Mice were tested according to the “Up and Down Method”, where the behavioral response of each animal to a single drug dose determined the drug dose administered to the next animal (and is increased or decreased by a predetermined log dose) [30]. Testing was carried out as described in Ozburn et al. 2010 with a starting dose of 2.5 g/kg ethanol [34]. Animals were maintained on a reverse light cycle and pseudo-randomly assigned to either the ZT3 or ZT15 LORR ED_50_ testing group (*n* = 12–13/ZT/genotype/sex). Each mouse was injected with a single dose of ethanol and tested only once. If a mouse did not display a LORR lasting 1 min, the ethanol dose for the next mouse would increase by a log interval of 0.025. If the mouse showed a LORR lasting at least 1 min, the ethanol dose for the next mouse would decrease by a log interval of 0.025. The ED_50_ values were determined by the following equation:

(1) $$ED_{50}\;=\;\frac{\sum x_{i}}{N}+\left(\frac{d}{N}\right)*\left(A+C\right)$$

where *x_i_* are the test levels, *N* = the last *N* trials, *d* = dosing interval, and *A* and *C* are constants listed in Table S2 from Dixon [30]. The 95% CI was determined using the following equation:

(2) $$95\%\;CI\;=\;\pm \left[d*\sqrt{\frac{2}{N}}*1.96\right]$$

where 1.96 reflects the 0.05 α level [30].

### 2.6. Operant Ethanol Self-Administration

We next tested whether selection altered ethanol consumption, cue seeking, and/or motivation for ethanol using operant oral ethanol self-administration procedures. We assessed latency to acquire lever pressing behavior to receive a food pellet reinforcer (food training), and behaviors during fixed ratio responding (FR1, FR3) for access to 20% ethanol. Mice were then subjected to extinction and cue-induced reinstatement testing. One cohort was subjected to an additional round of extinction cue-induced reinstatement testing, and another cohort was subjected to additional FR3 sessions followed by testing in a progressive ratio schedule of reinforcement for access to ethanol (PR testing). A total of 96 mice [2 cohorts of 48 mice (*n* = 8/genotype/sex/cohort)] were run through the entire operant self-administration experiment.

Mice were habituated to individual housing and sipper bottles for one week prior to testing. All operant testing chambers (Med Associates, VT, USA) were housed in light- and sound-attenuating boxes, and data were recorded using MedPC IV software (Med Associates, VT, USA). Figure 4a provides a visual representation of the testing chambers. Each chamber was outfitted for lever responding for food (trough) or liquid (sipper) reinforcement: one food pellet dispenser, trough for food pellet delivery, one sipper extender (outside the box unless extended) connected to a lickometer, two levers with cue lights above, and one house light. House light remained on during testing, except when indicated. Two separate cohorts of animals underwent the operant self-administration testing, as indicated in the following sections. The experimental timeline for each cohort is shown in Figure 4b.

### 2.7. Food Pellet Training

To ensure that mice learned how to lever press for access to a reinforcer, they first underwent food training. In each cohort, mice were weighed to determine their free feeding weight (FFW), then food restricted while in the home cage to maintain body weight at ~85% FFW for the duration of food training. After one day of food restriction, mice were placed individually in an operant testing chamber for a 1 h session. A single active (left) lever press resulted in the illumination of the cue light located above that lever, and mice received one reinforcer (food pellet, released into a trough on the opposite wall). Inactive (right) lever presses were recorded, but not accompanied by cue light illumination, and had no consequence. A successful trial was defined as earning 25 or more pellets in a 60 min session. If a mouse received 30 pellets, the session would end (the house light would turn off and no further lever presses would yield any food pellets). Endpoint criteria for the food training phase was defined as achieving three days of successful trials, after which mice were removed from further food training, and given free access to food for the remainder of the study. Three (out of 96) mice failed to achieve successful food training and were eliminated from further study. We confirmed that all mice returned to their free feeding weight prior to beginning the ethanol FR1 sessions.

### 2.8. Ethanol Self-Administration under a Fixed Ratio Schedule of Reinforcement (FR1/FR3)

Following food pellet training, both cohorts of mice were tested for acquisition of ethanol self-administration. Monday through Friday, mice were tested in 2 h sessions where an active (right) lever press resulted in a cue light above the lever and the delivery of 20% ethanol for 30 s (one access period; one reinforcer earned). After the access period, the house light went off for 8 s while the sipper retracted. Inactive (left) lever presses were recorded without consequence. No food was provided in the testing chamber. After 10 sessions (two weeks of FR1), mice were subjected to 10 FR3 sessions (for two weeks), where 3 active (right) lever presses were required for a single reinforcer.

### 2.9. Extinction 1/Reinstatement 1

Following FR3, both cohorts of mice were subjected to 5 daily 2 h sessions of extinction training, whereby lever pressing no longer resulted in reinforcer delivery or illumination of the cue light (the house light remained on; Extinction). Mice were then tested in a 1 day, 2 h reinstatement assay, in which an active (right) lever press resulted in a 30 s illumination of cue light. This cue-induced reinstatement of lever pressing is a model for seeking behavior.

### 2.10. Extinction 2/Reinstatement 2

The first cohort of mice underwent a second round of extinction and reinstatement immediately after Reinstatement 1 (Extinction 2/Reinstatement 2) because we did not observe a reduction in responding during extinction or an increase in responding during reinstatement. Here, we exposed mice to two 5 h long extinction sessions to test whether longer sessions would enable expression of expected extinction and reinstatement behaviors. We then tested mice in a second reinstatement session (as above).

### 2.11. Progressive Ratio

The second cohort of mice were not subjected to Extinction 2/Reinstatement 2: instead, following Reinstatement 1, we re-established FR3 responding for 30 s access to 20% ethanol (5 daily 2 h sessions). The following week, mice were tested on a 4 h PR schedule of reinforcement, where an increasing number of active (right lever; previously associated with ethanol access during FR1 and FR3) lever presses were required for each subsequent ethanol sipper reinforcer access and illumination of the cue light. The number of presses required is determine by the following equation:

(3) $$a_{n}=\;\frac{1}{8}*\left(2n^{2}+{\left(-1\right)}^{n}+7\right)$$

(where the pattern is 1, 2, 3, 5, 7, 10, 13, 17, 21, 26, etc.). Inactive (left) lever presses were recorded without consequence.

### 2.12. Statistics

Data are reported as the mean ± SEM value, except for LORR ED_50_ data, where values obtained from the “Up and Down Method” are reported as the ED_50_ ± 95% CI. Data were analyzed using one-, two- or three-way analyses of variance (ANOVAs), followed by post-hoc tests where appropriate. Where there were no significant sex interactions, data were collapsed on this factor. The one-, two-, and three-way ANOVA data analyses were performed, and graphs were generated, using GraphPad Prism 9 (GraphPad Software, La Jolla, CA, USA). The four-way repeated-measures ANOVAs of data were performed using SYSTAT 13.1 (Systat Software, Inc., San Jose, CA, USA). Figure 4 was created using BioRender software (https://biorender.com/ (accessed on 3 February 2021)).

## 3. Results

### 3.1. Drinking in the Dark (DID)/Drinking in the Light (DIL)

**Figure 1:**
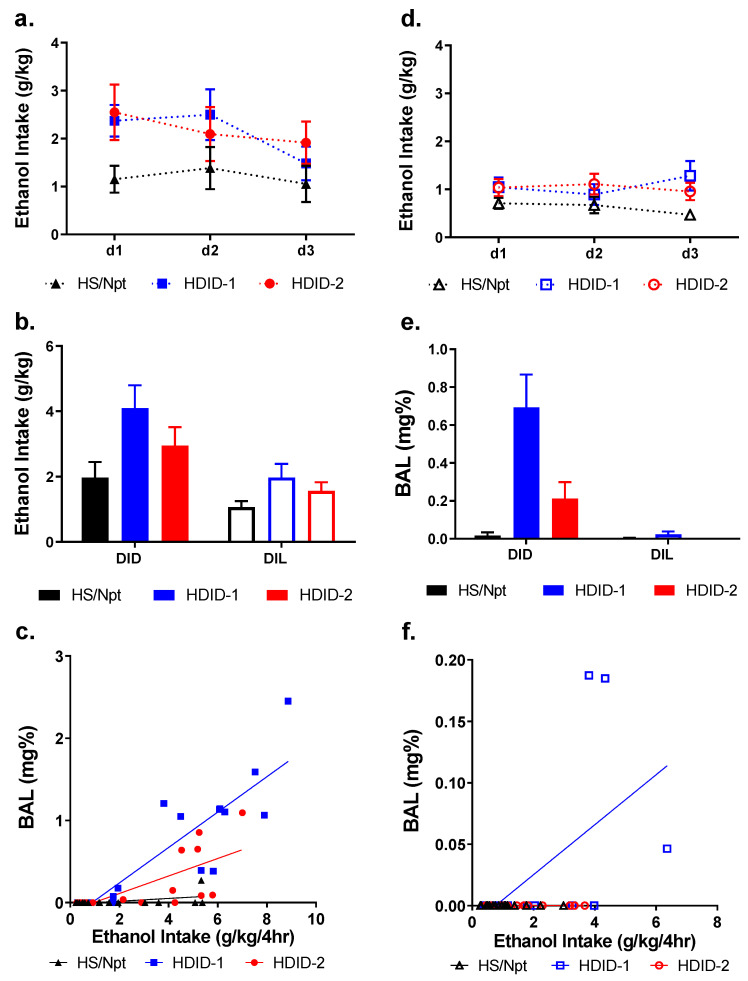
Ethanol consumption and achieved blood alcohol levels for Hs/Npt, HDID-1, and HDID-2 mice in Drinking in the Dark and Drinking in the Light assays. Mice were tested over four days for ethanol consumption and resulting BALs in both the dark and the light. Ethanol intakes for days 1–3, 2 h each day, are shown for drinking in the dark (**a**) and drinking in the light (**d**). Day 4 consisted of 4 h ethanol access: ethanol intake for drinking in both the dark and light (**b**) and resulting BALs (**e**) are shown. Also shown are graphs of the day 4 ethanol intake vs. BAL for the dark (**c**) and light (**f**). Sexes are collapsed for each genotype.

To test the hypothesis that the HDID selected lines would exhibit higher binge-like drinking than their progenitor line and that ethanol intake would show diurnal variation with increased intake occurring in the dark, we performed DID and DIL behavioral assays. A four-way repeated-measures ANOVA of the first three days of this experiment revealed significant main effects of sex [F > M; F(1, 87) = 5.93, *p* < 0.05], genotype [HDIDs > Hs/Npt; F(2, 87) = 4.16, *p* < 0.05], and time of day [dark > light; F(1, 87) = 17.19, *p* ≤ 0.0001]. There were no significant between-group interactions or interactions of day with other factors [Fs(2–4, 174) < 1.00], including sex, so the data are presented with the sexes collapsed for each genotype (Figure 1a,d) in the main paper, and separately for each genotype in Supplementary Materials Figure S1.

A three-way ANOVA of the ethanol intakes on the fourth day of this experiment revealed significant main effects of sex [F > M; F(1, 87) = 6.85, *p* ≤ 0.01], genotype [HDIDs > Hs/Npt; F(2, 87) = 4.99, *p* < 0.01], and time of day [dark > light; F(1, 87) = 14.88, *p* < 0.001]. However, there were no significant interactions [Fs(1–2, 87) < 1], so the data are presented with the sexes collapsed within genotype (Figure 1b).

A three-way ANOVA of the BALs on the last day revealed main effects of genotype [HDIDs > Hs/Npt; F(2, 87) = 9.10, *p* < 0.001] and of time of day [dark > light; F(1, 87) = 18.79, *p* < 0.0001], but not of sex [F(1, 87) < 1.00]. There was an interaction of genotype with time of day [F(2, 87) = 7.83, *p* < 0.001], but there were no other significant interactions [Fs(1–2, 87) < 1]. Bonferroni’s post-hoc revealed that HDID-1 BALs were significantly higher in DID than DIL [*p* < 0.0001], while HDID-2 [*p* = 0.3438] and Hs/Npt [*p* > 0.9999] were not significantly different across time. There were no significant interactions with sex, so the data are presented with the sexes collapsed within genotype (Figure 1e).

We determined the correlation between the ethanol intakes and resultant BALs both in the dark (Figure 1c) and light (Figure 1f) for the Hs/Npt, HDID-1, and HDID-2 mice. Regression lines for each genotype are significantly different, both in the dark [F(2, 44) = 11.82, *p* < 0.0001; r^2^(HDID-1) = 0.7518; r^2^(HDID-2) = 0.4975; r^2^(Hs/Npt) = 0.2236] and in the light [F(2,43) = 3.58, *p* < 0.05; r^2^(HDID-1) = 0.3283; r^2^(HDID-2) = 1.0; r^2^(Hs/Npt) = 1.0]. A notable limitation for the analysis of data in Figure 1f is that there are only three non-zero data points. Thus, the DIL BAL data lacks variability and likely does not provide a meaningful Pearson correlation value. What is perhaps more meaningful is that there were very few positive BALs for mice in the DIL group.

### 3.2. Relationship of DID and BAL over the 4 h DID Access Period

**Figure 2:**
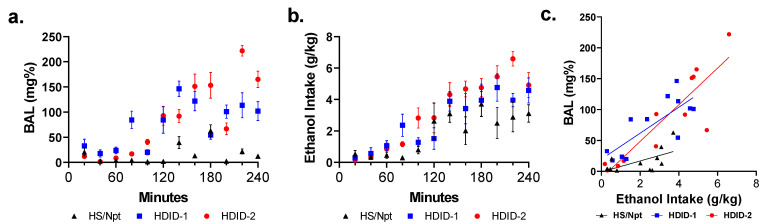
**Ethanol consumption and achieved blood alcohol levels at multiple time points during a 4 h Drinking in the Dark assay in Hs/Npt, HDID-1, and HDID-2 mice.** At 20 min increments during DID, a new set of animals was tested for ethanol intake (**a**) and blood was collected for BAL (**b**). Ethanol intake vs. BAL for each data point is plotted (**c**). HDID mice consumed more ethanol and reached higher BALs than Hs/Npt during DID. Differences in BAL are observed as early as 80 min (HDID-1>Hs/Npt and HDID-2), and 120 min (HDID-1 and HDID-2 > Hs/Npt) into DID (post-hoc testing results listed in Supplemental Table S2). The slopes of the HDID-1 and HDID-2 regression lines were steeper than the slope for Hs/Npt. No sex differences were observed, and sexes are collapsed within genotype.

We next tested separate groups of mice (*n* = 3/sex/genotype/time point) at 20 min intervals during the final day of a 4 day DID (4 h test day) to determine temporal drinking patterns and relate them to BALs achieved. The resulting ethanol consumptions and BALs over time are shown (Figure 2a,b). For ethanol intake and BAL data, two-way ANOVAs for each line revealed main effects for time (as expected), but no main effects of sex, and no sex x time interactions. Data were collapsed on sex for further analysis. Two-way ANOVA of ethanol intake (Figure 2a) revealed significant main effects of genotype [F(2163) = 14.51, *p* < 0.0001], and time [F(11,163) = 20.20, *p* < 0.0001], but not a genotype x time interaction [F(22,163) = 1.515, *p* = 0.07].

Two-way ANOVA of BALs (Figure 2b) revealed significant main effects of genotype [F(2177) = 107.1, *p* < 0.0001] and time [F(11,177) = 27.99, *p* < 0.0001], as well as genotype x time interaction [F(22,177) = 9.961, *p* < 0.0001]. Tukey’s post-hoc testing was carried out to test for genotype differences at each time point. Differences are observed as early as 80 min (HDID-1>Hs/Npt and HDID-2), and 120 min (HDID-1 and HDID-2 > Hs/Npt) into DID. A full listing of post-hoc testing is presented in Supplemental Table S2.

Also shown are the ethanol intakes versus BAL for the same time points (Figure 2c). Regression lines for each genotype are significantly different [F(2, 30) = 3.842, *p* < 0.05; r^2^(HDID-1) = 0.6047; r^2^(HDID-2) = 0.7829; r^2^(Hs/Npt) = 0.3703]. The slopes of the HDID-1 and HDID-2 regression lines were steeper than the slope for Hs/Npt.

### 3.3. Loss of Righting Reflex Diurnal ED_50_

**Figure 3:**
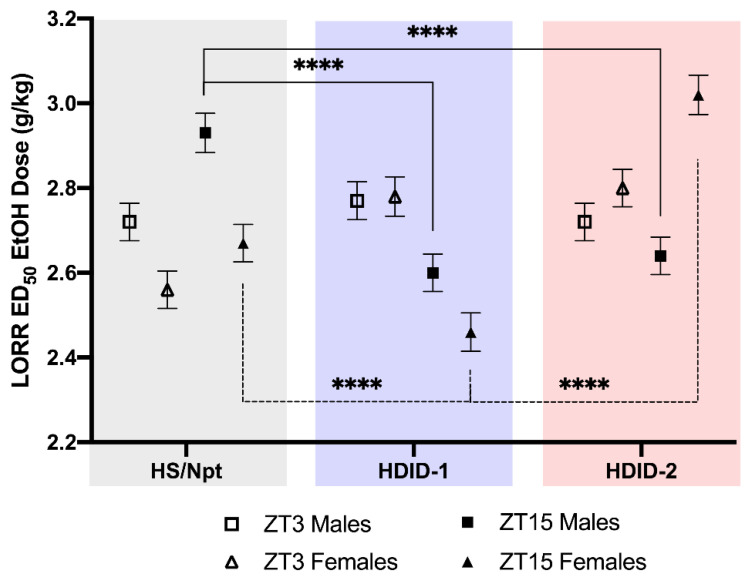
**HDID-1, HDID-2, and Hs/Npt mice exhibit differential diurnal sensitivity to the sedative effects of ethanol.** We determined the ethanol ED_50_ for LORR in both the light and dark for males and females of each genotype and observed a significant genotype x sex x ZT interaction [F (2, 120) = 37.16]. Select Tukey’s post-hoc results are shown here to illustrate genotype differences at ZT15, when highest levels of drinking have been observed. Male (closed squares) HDID-1 and HDID-2 mice have a lower ED_50_ than Hs/Npt at ZT15, indicating that they are more sensitive to the sedative effects of ethanol. At ZT15, female (closed triangles) HDID-1 have a lower ED_50_ than Hs/Npt (more sensitive to ethanol), whereas female HDID-2 have a higher ED_50_ than Hs/Npt (less sensitive). **** *p* < 0.0001. Additional post-hoc testing results are provided in Supplemental Table S1. Data presented as ED_50_ with the 95% CI.

To test the hypothesis that the sensitivity to the sedative/hypnotic effects of ethanol is dependent on time of day and genotype, we determined the effective dose of ethanol required for 50% of the population (ED_50_) to lose righting reflex for each genotype at ZT3 (inactive/light cycle) and ZT15 (active/dark cycle) (Figure 3). Larger ED_50_ values indicate less sensitivity to ethanol’s sedative effects. We collected ZT3 and ZT15 data for each genotype within a single day, and each genotype was tested on a separate day (a limitation noted here).

Results of the three-way ANOVA revealed a significant main effect of genotype [F(2, 120) = 48.84, *p* < 0.0001], and genotype x ZT [F(2, 120) = 107.6, *p* < 0.0001] and genotype x sex [F(2, 120) = 124.5, *p* < 0.0001] interactions, as well as a genotype x sex x ZT interaction [F(2, 120) = 37.16, *p* < 0.0001]. Tukey’s post-hoc analysis was carried out to assess the effect of (1) genotype differences for each sex, within each ZT (e.g., compare ZT3 for female Hs/Npt and female HDID-1 mice); (2) sex at each ZT, within each line (e.g., compare male and female Hs/Npt at ZT3); and (3) ZT for each sex, within each line (e.g., compare ZT3 and ZT15 for female Hs/Npt mice).

Interesting results include genotype differences for each sex (comparison 1, above), and are highlighted for ZT15 in Figure 3. We first focus on ZT15 because that is when DID begins, and when mice have been shown to drink to intoxication. Male HDID-1 and HDID-2 mice have a lower ED_50_ than Hs/Npt at ZT15 (*p* < 0.0001 for both), indicating that they are more sensitive to the sedative effects of ethanol. At ZT15, female HDID-1 have a lower ED_50_ than Hs/Npt (*p* < 0.0001, more sensitive to ethanol), whereas female HDID-2 have a higher ED_50_ than Hs/Npt (*p* < 0.0001, less sensitive). However, at ZT3, in females we observe that HDID-1 and HDID-2 have a higher ED_50_ that Hs/Npt (*p* < 0.0001 for both, suggesting that females of selected lines are less sensitive). No genotype difference was observed in males at ZT3.

We assessed whether sex differences were observed at each ZT within each line (comparison 2, above). For Hs/Npt, we observed sex differences at ZT3 (*p* < 0.0001) and ZT15 (*p* < 0.001), and for HDID-1, we observed sex differences at ZT15 (*p* < 0.001), where females were more sensitive to the sedative effects of ethanol. For HDID-2, we observed a sex difference at ZT15 (*p* < 0.001), where females were less sensitive to ethanol.

We assessed whether ZT differences were observed for each sex, within each line (comparison 3, above). Hs/Npt males (*p* < 0.0001) and females (*p* < 0.01) both show diurnal variation in ED_50_, where sensitivity is lower during the dark (ZT15). HDID-1 males (*p* < 0.0001) and females (*p* < 0.0001) both show diurnal variation in ED_50_, where sensitivity is higher during the dark (ZT15). HDID-2 females (*p* < 0.0001) show diurnal variation in ED_50_, where sensitivity is lower during the dark (ZT15).

### 3.4. Operant Self-Administration: Food Training

**Figure 4:**
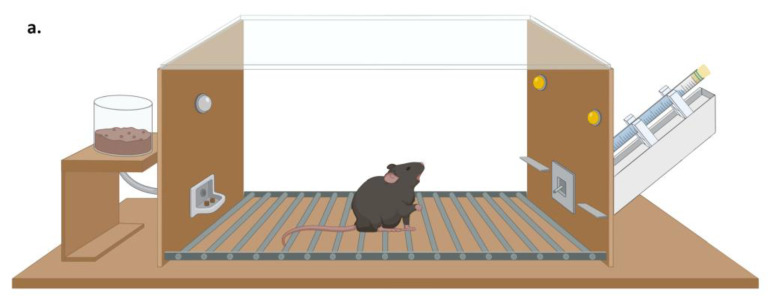

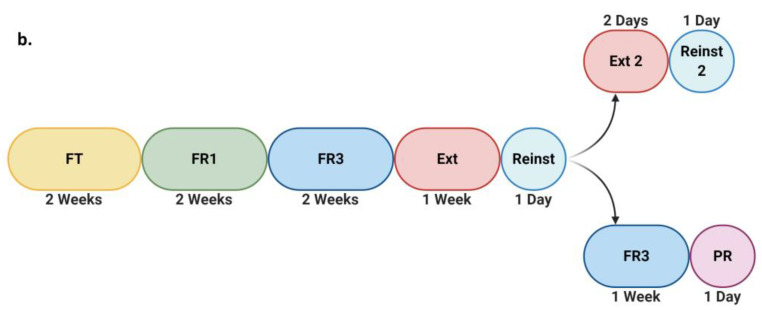
**Schematic of operant chamber and experimental timelines for operant ethanol self-administration experiment.** Operant chambers were contained in light- and sound-attenuating boxes. Chambers contained a food trough and house light on one chamber wall, and two levers with associated cue lights and extendable/retractable fluid sipper on the opposite chamber wall (**a**). Two cohorts of mice underwent two weeks of food training (FT), followed by two weeks of FR1 testing, two weeks of FR3, one week of Extinction (Ext), and one day of Reinstatement (Reinst). After this, one cohort underwent a second Extinction (Ext 2) and Reinstatement (Reinst 2) test, while the other cohort underwent a week of FR3, followed by a day of PR testing (**b**).

To test the hypothesis that HDID lines will demonstrate greater motivation for ethanol than their founders as measured by operant behaviors, we conducted an operant oral ethanol self-administration experiment in two cohorts. Figure 4a shows unique features of the operant chambers used here and Figure 4b illustrates the experimental timelines. All mice first underwent a standard food training procedure to acquire an association between cue, lever press, and a food pellet reinforcer.

**Figure 5:**
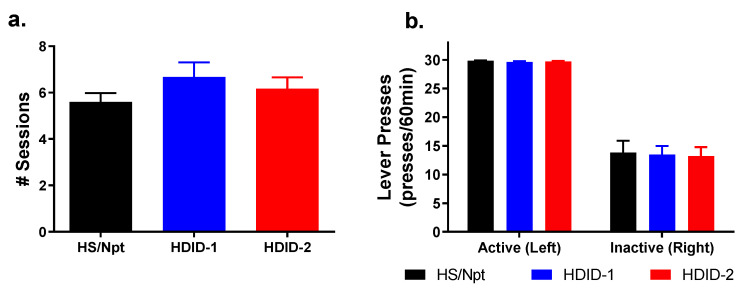
**Hs/Npt, HDID-1, and HDID-2 mice exhibit similar operant chow pellet self-administration behaviors.** No observed genotypes differenced in meeting criteria for operant self-administration of food pellets (food training). Shown are the latency to meet criteria among Hs/Npt, HDID-1, and HDID-2 mice (**a**), and the active and inactive lever presses (**b**). Sexes are collapsed within genotypes.

A two-way ANOVA of the latency to meet food training criterion revealed a significant main effect of sex [F(1,82) = 6.958, *p* = 0.01], but not an effect of genotype [F(2,82) = 1.319, *p* = 0.2730], and no significant interaction [F(2,82) = 0.5506, *p* = 0.5787]. As no significant interaction was present, data are shown with sex collapsed within genotype (Figure 5a).

Data on the active and inactive lever presses for the successful tests are also shown (Figure 5b) and demonstrate that mice were learning the association by engaging in more active lever pressing than inactive lever pressing.

### 3.5. Operant Self-Administration: FR1

**Figure 6:**
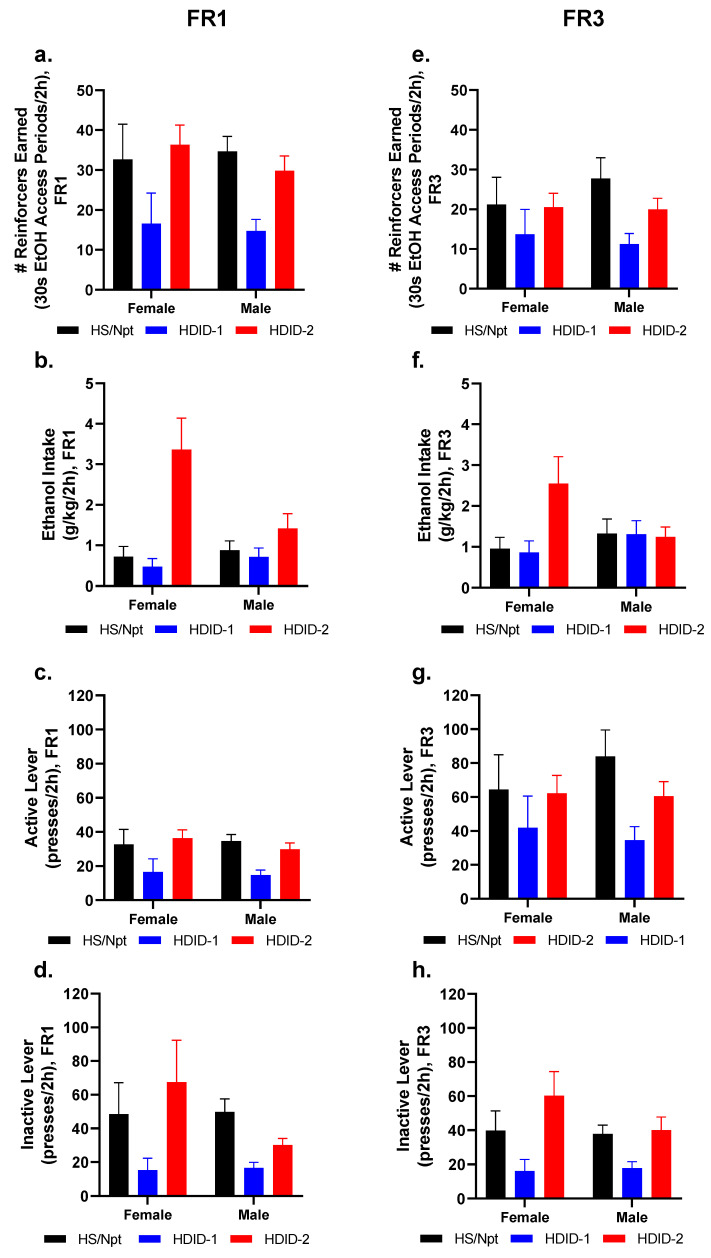
**Differential operant ethanol self-administration behaviors observed in Hs/Npt, HDID-1, and HDID-2 mice.** Mice were tested for operant 20% ethanol self-administration under an FR1 (**a**–**d**) and FR3 (**e**–**h**) schedules of reinforcement. Average data are shown for the last three days in each FR schedule for the number of reinforcers (30 s access to ethanol sipper) earned (**a**,**e**), ethanol intake (**b**,**f**), active lever presses (**c**,**g**) and inactive lever presses (**d**,**h**) per session. For complete data, see Supplemental Figure S2.

After food training, mice were trained to lever press for an ethanol reinforcer [30 s access to sipper tube containing 20% ethanol (as used in DID)] under an FR1 schedule (Figure 4b). While all data were collected and can be viewed in Supplemental Figure S2, Figure 6 shows the average of the final three days of FR1 testing for each sex and genotype, with regards to the number of reinforcers (30 s ethanol access periods) earned (Figure 6a), ethanol intake (Figure 6b), active lever presses (Figure 6c), and inactive lever presses (Figure 6d).

Two-way ANOVA analysis of the average number of access periods during the final three days of FR1 testing revealed a significant effect of genotype [F(2, 84) = 6.289, *p* < 0.01], with the HDID-1 animals receiving significantly fewer access periods than the other genotypes. No significant effect of sex [F(1, 84) = 0.1956, *p* = 0.66] was found, and there was no significant genotype by sex interaction [F(2,84) = 0.2731, *p* = 0.76] (Figure 6a).

A two-way ANOVA of the average ethanol intake during the final three days of FR1 testing revealed a significant effect of genotype [F(2, 84) = 11.49, *p* < 0.0001], but not sex [F(1, 84) = 2.364, *p* = 0.13]. A significant interaction was detected between genotype and sex [F(2, 84) = 4.608, *p* < 0.05]. Tukey’s multiple comparisons revealed that the female HDID-2 mice had significantly higher intake than either the HDID-1 [*p* < 0.0001] or Hs/Npt [*p* < 0.0001] females - the males, however, were not significantly different (Figure 6b).

A two-way ANOVA of the average number of active lever presses during the final three days of FR1 testing revealed a significant effect of genotype [F(2, 84) = 6.28, *p* < 0.01], with the HDID-1 mice pressing the active lever significantly less than the other genotypes. There was no significant effect of sex [F(1, 84) = 0.66] and no significant interaction [F(2, 84) = 0.2705, *p* = 0.76] (Figure 6c).

A two-way ANOVA of the average number of inactive lever presses during the final three days of FR1 testing revealed a significant effect of genotype [F(2, 84) = 3.671, *p* < 0.05], with the HDID-1 animals pressing the inactive lever less than the other genotypes. There was no significant effect of sex [F(1, 84) = 1.03, *p* = 0.31] and no significant interaction [F(2, 84) = 1.25, *p* = 0.29] (Figure 6d).

### 3.6. Operant Self-Administration: FR3

Following FR1 testing, the mice were subjected to FR3 ethanol testing (Figure 4b). Just as in the FR1 section above, all data were collected and can be viewed in Supplemental Figure S2, while Figure 6 shows the average of the final three days of FR3 testing for each sex and genotype, with regards to the number of reinforcers (30 s ethanol access period) earned (Figure 6e), ethanol intake (Figure 6f), active lever presses (Figure 6g), and inactive lever presses (Figure 6h).

A two-way ANOVA of the average number of access periods during the final three days of FR3 testing revealed a significant effect of genotype [F(2, 84) = 3.173, *p* < 0.05], with the HDID-1 mice receiving fewer access periods than the Hs/Npt mice. There was no significant effect of sex [F(1, 84) = 0.08870, *p* = 0.77] and no significant interaction [F(2, 84) = 0.4761, *p* = 0.62] (Figure 6e).

A two-way ANOVA of the average ethanol intake over the last three days of FR3 revealed that the effects of genotype approached but did not achieve significance [F(2, 84) = 2.576, *p* = 0.0821], and there was no effect of sex [F(1, 84) = 0.2598, *p* = 0.61]. The interaction of genotype x sex also approached but did not achieve significance [F(2, 84) = 3.096, *p* = 0.0504] (Figure 6f).

A two-way ANOVA of the average number of active lever presses during the final three days of FR3 testing revealed a significant effect of genotype [F(2, 84) = 3.159, *p* < 0.05], with the HDID-1 animals pressing the active lever less than the Hs/Npt animals. There was no effect of sex [F(1, 84) = 0.08711, *p* = 0.77] and the interaction of genotype by sex was not significant [F(2, 84) = 0.4690, *p* = 0.63] (Figure 6g).

A two-way ANOVA of the average number of inactive lever presses during the final three days of FR3 testing revealed a significant effect of genotype [F(2, 84) = 6.845, *p* < 0.01], with the HDID-1 mice pressing the inactive lever less than the HDID-2 mice. There was no significant effect of sex [F(1, 84) = 0.8360, *p* = 0.36] and no significant genotype by sex interaction [F(2, 84) = 0.8272, *p* = 0.44] (Figure 6h).

### 3.7. Extinction 1/Reinstatement 1

To test ethanol-seeking behavior, we subjected all mice to extinction and cue-induced reinstatement testing (Figure 4b).

**Figure 7:**
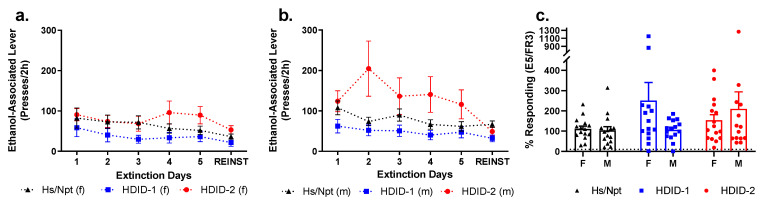
**Hs/Npt, HDID-1, and HDID-2 mice behaviors under extinction and cue-induced reinstatement conditions.** Mice underwent five sessions of extinction and one session of reinstatement. Data for each genotype’s pressing of the “active” (previously ethanol-associated, now inactive) lever for females (**a**) and males (**b**) is shown here. Also shown is the % responding (for ethanol-associated lever) for the final day of Extinction compared to the average of the last three days of FR3 testing (**c**). For graphs on pressing of the “inactive” lever (formerly food-associated), see Supplemental Figure S3.

A repeated-measures, two-way ANOVA of the number of right (formerly ethanol-associated) lever presses during the final day of extinction revealed a main effect of genotype [F(2, 84) = 5.379, *p* < 0.01], with the HDID-1 mice pressing significantly less than the HDID-2. There was no significant effect of sex [F(1, 84) = 1.006, *p* = 0.32] and no significant interaction of genotype by sex [F(2, 84) = 0.1146, *p* = 0.89] (Figure 7a,b).

For each mouse, we also compared the percent response for ethanol-associated lever presses on the final day of Extinction compared to the average of the final three days of FR3 testing (Figure 7c). Data points more than two standard deviations above the mean were omitted as outliers. No significant differences between sexes or among genotypes were observed [*p* > 0.13].

Because mice did not extinguish ethanol-seeking behaviors (as would be evidenced by diminished lever pressing of the formerly active lever to less than 10% of responding when the lever was active), we did not analyze the reinstatement data, but present it here for transparency (Figure 7a,b).

### 3.8. Extinction 2/Reinstatement 2

**Figure 8:**
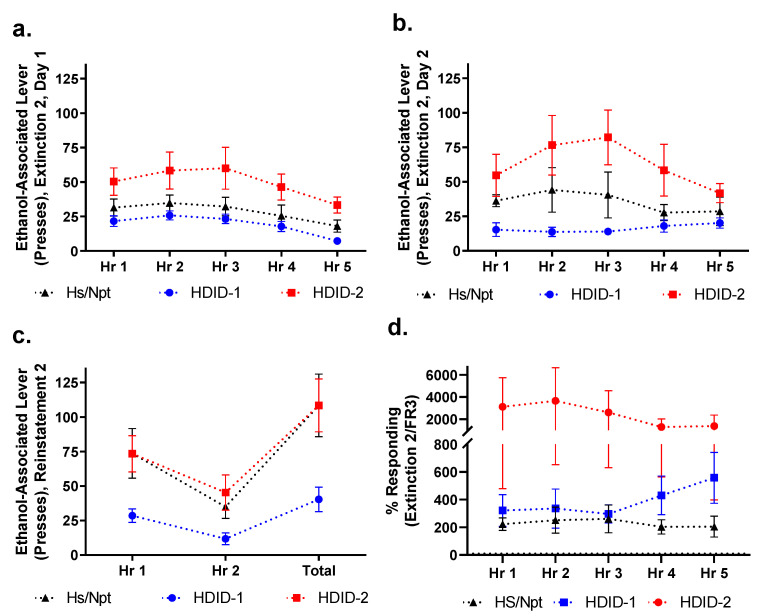
**Hs/Npt, HDID-1, and HDID-2 mice behaviors under additional extinction and cue-induced reinstatement conditions.** Mice underwent Extinction 2 and Reinstatement 2. Data shown here are the hourly breakdowns of the formerly ethanol-associated lever presses for Extinction 2 Day 1 (**a**), Extinction 2 Day 2 (**b**), and Reinstatement 2 (**c**). Also shown is a comparison of the hourly lever presses of the formerly ethanol-associated lever on Extinction 2 Day 2 to the average active presses per hour across the final three days of FR3 (**d**). Sexes are collapsed within genotypes. See Supplemental Figure S4 for results on the formerly food-associated lever presses.

In order to test whether the mice were capable of extinction if given a longer session, the first cohort of operant mice were put through a second set of extinction and reinstatement, consisting of two, 5 h sessions of extinction (Extinction 2), and a single, 2 h reinstatement (Reinstatement 2) (Figure 4b). Data shown are the average number of right (formerly ethanol-associated) lever presses during each hour of Extinction 2 Day 1 (Figure 8a), Extinction 2 Day 2 (Figure 8b), and Reinstatement 2 (Figure 8c).

As with Extinction 1, we compared the final day of Extinction 2 to the average data from the FR3 testing. Here we compared the number of formerly ethanol-associated lever presses for each hour (non-cumulative) during Extinction 2 Day 2 to the average per hour lever presses for the final three days of FR3. A repeated-measures, two-way ANOVA of each of these percentiles revealed a main effect of genotype [F(2, 210) = 5.161, *p* < 0.01], but not of time [F(4, 210) = 0.2273, *p* = 0.92] and no significant interaction [F(8, 210) = 0.2759, *p* = 0.97] (Figure 8d). Again, we saw that the mice did not meet extinction criterion, so no analysis was performed on Reinstatement 2 (Figure 8c).

### 3.9. Progressive Ratio

To test whether selection altered the motivation for ethanol self-administration, the second cohort of operant mice underwent a single, 4 h progressive ratio session (Figure 4b).

**Figure 9:**
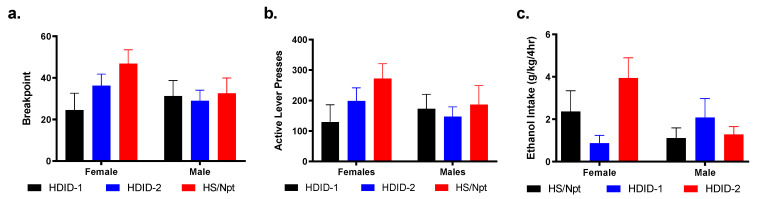
**Hs/Npt, HDID-1, and HDID-2 mice show similar motivation for access to ethanol but drink different amounts of ethanol.** Mice underwent a self-administration progressive ratio test, where each successive reinforcer requires more active presses. Graphs shown here are the breakpoint (**a**), the total number of active presses (**b**), and the ethanol intake (**c**).

A two-way ANOVA analysis of the breakpoints revealed no significant effects of genotype [F(2, 39) = 1.508, *p* = 0.23] or sex [F(1, 39) = 0.7629, *p* = 0.39], and no significant interaction [F(2, 39) = 1.214, *p* = 0.31] (Figure 9a).

Likewise, a two-way ANOVA analysis of the total active presses revealed no significant effects of genotype [F(2, 39) = 1.276, *p* = 0.29], sex [F(1, 39) = 0.5811, *p* = 0.45], or genotype by sex interaction [F(2, 39 = 0.8763, *p* = 0.42] (Figure 9b).

A two-way ANOVA analysis of the ethanol intake revealed no significant effects of genotype [F(2, 39) = 1.381, *p* = 0.26] or sex [F(1, 39) = 2.358, *p* = 0.13], but did reveal a significant genotype by sex interaction [F(2, 39) = 3.697, *p* < 0.05]. Tukey’s post-hoc analysis revealed that HDID-2 female mice consumed more ethanol on average than the HDID-1 female mice [*p* < 0.01] (Figure 9c).

## 4. Discussion

The genetic contribution to AUD is well known yet identifying important mechanisms remain challenging. A major barrier in this pursuit is the complexity of AUD, which includes a web of factors, such as complex genetic, environmental, and gene-by-environment interactions, that increase risk for developing an AUD [15]. Although the use of human genome-wide association studies (GWAS) have proven beneficial for the alcohol field—as well as many other mental health fields—they remain challenged by the need for large sample sizes and the difficulty in fully estimating trait heritability [35,36,37]. By testing known genetic and behavioral endophenotypes of AUD across several behavioral and physiological measures of AUD risk, we can develop a framework to better identify key mechanisms and treat this disease. Here we report the findings of testing replicate lines of HDID mice, compared to their heterogenous founders, across the following important AUD-related measures: diurnal and temporal limited-access ethanol intake, sensitivity to the sedative effects of ethanol, cue-induced ethanol seeking and the motivation to drink ethanol. We found that HDID lines drink more ethanol than Hs/Npt during the dark and light in limited-access paradigms, and that all lines drink more during the dark than in the light. We thought that this might reflect differential sensitivity to ethanol and expected that mice might be less sensitive to the sedative effects of ethanol in the dark. While Hs/Npt and HDID-2 mice were less sensitive to ethanol during the dark, HDID-1 mice were not. Moreover, we hypothesized that HDID lines would exhibit greater motivation for ethanol, yet their “breakpoints” do not differ from their genetically heterogeneous founders, Hs/Npt. Additional findings of note include distinct strain and sex differences among some of these behaviors and are discussed further below.

As expected, HDID-1 and HDID-2 mice drank significantly more ethanol than Hs/Npt during both DID and DIL tests. Across all three strains, mice drank more during DID than DIL, and pharmacologically relevant BALs were not achieved during the DIL task. This is consistent with the known increase in consumptive behavior of rodents during the dark cycle and has been part of the rationale for using nocturnal limited ethanol access to increase intake and model binge-like ethanol drinking [18,38,39,40]. On average, HDID and Hs/Npt mice drank ~1–2 g/kg of ethanol during the 4 h DIL, which is comparable to baseline C57BL6/J male intake prior to chronic intermittent ethanol exposure (CIE) paradigm, in which ethanol is offered for 2 h at the end of the light cycle [41,42,43]. While testing during a similar CIE procedure (in which ethanol is offered at the end of the light cycle), HDID-1 and Hs/Npt drank comparable amounts of ethanol during DIL [10]. HDID-1 mice drank significantly more than Hs/Npt during baseline (similar to what we observed), despite showing equivalent drinking escalations during withdrawal [10]. Taken together, these findings suggest that selection for drinking to intoxication in the dark similarly increased ethanol intake in the light (during limited-access drinking sessions).

A major factor in reaching intoxicating BALs within a drinking session is the pattern of intake, whereby the size and number of drinking bouts have been associated with the risk for heavy drinking [44,45,46]. Work by Grant et al. has shown that large bouts over a short duration (a ‘gulping’-like phenotype) was associated with the development of heavy drinking in non-human primates [47]. HDID-1 mice show no difference compared to Hs/Npt in drinking microstructure or total consumption in continuous, 2-bottle choice drinking, but do show a higher ethanol preference [48]. In contrast, during both a 2 and 4 day DID, HDID-1 mice displayed more frequent drinking bouts, shorter interbout intervals, and larger bout sizes compared to Hs/Npt, despite reaching moderately high intake and BAL [48]. Subsequent analysis of the pattern and microstructure during DID in Hs/Npt, HDID-1, and HDID-2 mice revealed that the two HDID replicate lines reach intoxicating BALs through different means. HDID-1 mice drink in larger bout sizes, while HDID-2 drink in smaller more frequent bouts, much like Hs/Npt. However, Hs/Npt do not drink enough to reach intoxicating BALs [22]. Whereas C57BL6/J mice display “front -loading” behavior, in which more ethanol is consumed during the early part of DID, HDID mice have been shown to drink consistently high amounts throughout either 2 or 4 h drinking sessions [22]. Here we report intake and subsequent BALs at 20 min intervals during a 4 h DID in Hs/Npt, HDID-1, and HDID-2 mice of both sexes. All three genotypes showed steady increases in ethanol intake throughout the 4 h session. We saw a comparable delay in the time to first peak of drinking, which occurred at approximately 80 min for HDID-1 and 100 min for the HDID-2. It took approximately 80 and 120 min for both HDID replicates to reach intoxicating BALs (>80% mg), whereas at no point did Hs/Npt exceed BALs >50 mg%. These data show that HDID mice will drink to intoxication in a 2 h session, and will continue in this excessive manner of drinking, achieving BALs that are >2–3× higher in the next 2 h. Moreover, HDID mice do not require excessively chronic drinking paradigms to demonstrate this front-loading pattern of drinking. Therefore, we tested whether HDID mice reach such high BALs because of an initial low level of response to alcohol.

Sensitivity to the intoxicating effects of ethanol is considered a determinant of alcohol intake and AUD risk and is thought to be in part genetically driven [49,50,51,52,53]. For example, Kurtz et al. compared the loss of righting reflex duration following a 3.0 g/kg ethanol injection in selectively bred alcohol-preferring (P) and -non-preferring (NP) rats and found that P rats were less sensitive to the ethanol-induced behavioral impairment [54]. Moreover, B6 x FVB F1 hybrid mice, which show sustained high ethanol intake and preference, are less sensitive to the sedative and aversive effects, but not the rewarding effects of ethanol than other B6 F1 hybrid mice which exhibit lower ethanol intake [34]. To determine whether ethanol sensitivity is a genetic correlate of selection for binge-like ethanol drinking, we determined the ethanol ED_50_ for LORR in Hs/Npt, HDID-1, and HDID-2 mice, a measure of sensitivity to the intoxicating and sedative effects of ethanol. To better understand ethanol sensitivity in relationship to time of day, mice were tested at two important circadian time points: ZT3 and ZT15 (which corresponds with the start of DIL and DID, respectively). For ZT15, we found that HDID-1 mice were more sensitive than Hs/Npt (require a lower dose of ethanol to achieve sedation), and that HDID-2 mice were less sensitive than Hs/Npt mice (require a higher dose of ethanol). These findings are similar to the duration of LORR (in response to a 4 g/kg dose), where HDID-1 mice exhibited a longer duration of LORR than Hs/Npt, and HDID-2 mice exhibited a shorter duration [11]. We observed interesting time of day effects, where Hs/Npt and HDID-2 mice exhibited lower sensitivity in the dark (higher doses of ethanol are required to achieve sedation). We found the opposite effect for HDID-1 mice, where they exhibited higher sensitivity during the dark (lower doses of ethanol are required to achieve sedation). We caution against interpreting these results as evidence that sensitivity to the sedative effects of ethanol is not genetically correlated with excessive alcohol drinking. The HDID-1 and HDID-2 selected lines were created independently [8,9] and are genetically distinct from each other, as well as their founder line [55]. Thus, HDID-1 and HDID-2 likely represent two different constellations of genes that contribute to risk for excessive ethanol drinking and may not show similar responses for other polygenetic traits. Lastly, sensitivity to the sedative effects of ethanol does not provide insight for the high intake seen during the dark and lower intake during the light in these mouse lines.

Ethanol seeking and craving are also critical components in risk for the development of AUD and the risk of relapse. When presented with an odor cue, non-dependent humans show higher subjective craving for alcohol [56]. Similarly, alcohol cues, including the presentation of an alcoholic beverage, have been shown to increase the subjective desire to drink in social drinkers and individuals with an AUD [57,58,59]. Moreover, ethanol seeking and craving are thought to be in part genetically derived [60,61,62]. Operant self-administration has been used to model ethanol seeking in rodents, wherein animals are exposed to ethanol-associated stimuli in the absence of response-contingent ethanol reinforcement. In this way, cue-induced responding can be used as a measure of ethanol-seeking behavior. As a limitation to the presented work, extinction criteria were not met among any of the genotypes (after five days of extinction, only 2 of the 93 mice tested achieved extinction criteria, and extinction criteria were still not met after 20 h of extinction). The lack of extinction in HDID and Hs/Npt mice is somewhat consistent with similar experiments using C57BL/6J mice, where >15 and 60 sessions are needed to reach extinction criteria after similar levels of ethanol self-administration [63,64]. The ability of a single stimulus, especially light or sound, to induce reinstatement in non-dependent animals is known to be limited [65,66]. Therefore, future studies would benefit from testing HDID and Hs/Npt mice using olfactory or a combination of cues to reliably reinstate ethanol seeking [65,67,68].

Lastly, we sought to test whether selecting for drinking to high BALs altered the motivation for ethanol by testing Hs/Npt, HDID-1, and HDID-2 mice under a progressive ratio schedule of reinforcement for access to 20% ethanol. It is well established that the motivation for ethanol is a central trait in AUD development, and that there is a genetic and likely neuromolecular basis for the gating of the motivational effects of ethanol [69,70,71,72]. The motivation for ethanol access can be inferred from operant responding under a PR schedule by determining the breakpoint, or the maximal number of responses given to receive ethanol access. Quite surprisingly, we found that Hs/Npt, HDID-1, and HDID-2 mice reached comparable breakpoints (and total reinforcers earned) in this motivational task, suggesting that there is no difference between these phenotypes in the motivation for access to ethanol. HDID-2 showed higher consumption than either Hs/Npt or HDID-1 mice during FR1 and FR3. Therefore, HDID-1 and Hs/Npt mice appear similar in their ethanol intake during operant self-administration (conducted at the same ZT as DID), despite having a known difference in DID ethanol intake. Throughout operant testing, the stark difference in binge-like ethanol drinking behavior was juxtaposed by the notable similarity between Hs/Npt and both HDID genotypes for operant responding. Though interesting, this suggests that further endophenotype testing is needed to better determine the potential genetic and behavioral differences between these genotypes. Ongoing studies will benefit from testing other indices of ethanol motivation, such as quinine-adulterated ethanol drinking, which has been used to assess the effects of devaluing the ethanol reinforcer, as well as address drinking despite negative consequences and habitual responding. Recent work by Sneddon et al. shows that female C57BL/6J mice habitually respond for 10% ethanol in an operant task, a concentration with no sex difference in baseline responding [73]. Given observed sex effects and sex x genotype interactions for diurnal and temporal drinking patterns during DIL and DID, we will address potential sex differences for habitual and motivational ethanol responding.

Our behavioral genetics interpretation is limited to the three genotypes studied and does not mean that sensitivity to the sedative effects of ethanol is unrelated to binge drinking. Data thus far suggest that different constellations of genes were selected for in HDID-1 and HDID-2 mice, so it is not really a surprise that they do not differ from Hs/Npt in the same way. This changes how confidently one can infer for each general trait (e.g., sedation) that some similar genes are involved in a generally important way. Additional preclinical and clinical studies are needed here for a better understanding of what role these phenotypes play across the spectrum of AUD models and AUD. Taken together, these findings suggest that selection for high BALs after DID is associated with differences in some, but not all, behavioral phenotypes of diurnal and temporal limited-access intake, sensitivity to the intoxicating effects of ethanol, ethanol seeking and the motivation for ethanol.

## Data Availability

Authors have full control of all primary data and agree to allow the journal and any subsequent readers of the published work to review the data if requested.

## Supplementary Materials

The following are available online at https://www.mdpi.com/2076-3425/11/2/189/s1, Supplemental Figure S1 (Ethanol consumption and achieved blood alcohol levels for Hs/Npt, HDID-1, and HDID-2 mice in Drinking in the Dark and Drinking in the Light assays: males and females shown separately), S2 (Differential operant ethanol self-administration behaviors observed in Hs/Npt, HDID-1, and HDID-2 mice: data for all sessions), S3 (Hs/Npt, HDID-1, and HDID-2 mice behaviors under extinction and cue-induced reinstatement conditions: previously food-associated lever presses (this lever was inactive during ethanol sessions)), S4 (Hs/Npt, HDID-1, and HDID-2 mice behaviors under additional extinction and cue-induced reinstatement conditions: previously food-associated lever presses (this lever was inactive during ethanol sessions)), and Supplemental Table S1 (Hs/Npt, HDID-1, and HDID-2 mice exhibit differential diurnal sensitivity to the sedative effects of ethanol: three-way ANOVA results) and S2 (Ethanol consumption and achieved blood alcohol levels at multiple time points during a 4 h Drinking in the Dark assay in Hs/Npt, HDID-1, and HDID-2 mice: Tukey’s post-hoc results for BAL analysis).
