# Supplementary material for: Ethanol-Related Behaviors in Mouse Lines Selectively Bred for Drinking to Intoxication
Source: Brain Sci. 2021 Feb 4;11(2):189. doi: 10.3390/brainsci11020189 (PMC7915226; doi:10.3390/brainsci11020189)
Supplement: Supplementary file 1 [file brainsci-11-00189-s001.pdf]

brain sciences

MDPI

Article

Ethanol-related behaviors in mouse lines selectively bred for drinking to intoxication: Supplemental Figures

Table S1. Hs/Npt, HDID-1, and HDID-2 mice exhibit differential diurnal sensitivity to the sedative effects of ethanol: three-way ANOVA results. Data presented in Figure 3. Red text indicates significant results of interest. * symbols in Summary column denotes Adjusted p Value shown in far-right column.

LORR ED_{50} 3-way ANOVA Table	F (DFn, DFd)	p value
Line	F (2, 120) = 48.84	P<0.0001
ZT	F (1, 120) = 0.1819	P=0.6705
Sex	F (1, 120) = 1.637	P=0.2032
Line x ZT	F (2, 120) = 107.6	P<0.0001
Line x Sex	F (2, 120) = 124.5	P<0.0001
ZT x Sex	F (1, 120) = 0.5053	P=0.4785
Line x ZT x Sex	F (2, 120) = 37.16	P<0.0001
**Tukey's multiple comparisons test**	**Summary**	**Adjusted p Value**
**Selected focus: within line, sex difference for each ZT**		
HS/Npt:ZT3 Females vs. HS/Npt:ZT3 Males	****	<0.0001
HS/Npt:ZT15 Females vs. HS/Npt:ZT15 Males	****	<0.0001
HDID-1:ZT15 Females vs. HDID-1:ZT15 Males	***	0.0002
HDID-2:ZT15 Females vs. HDID-2:ZT15 Males	****	<0.0001
**Selected focus: within line, ZT difference for each sex**		
HS/Npt:ZT3 Females vs. HS/Npt:ZT15 Females	**	0.0053
HS/Npt:ZT3 Males vs. HS/Npt:ZT15 Males	****	<0.0001
HDID-1:ZT3 Females vs. HDID-1:ZT15 Females	****	<0.0001
HDID-1:ZT3 Males vs. HDID-1:ZT15 Males	****	<0.0001
HDID-2:ZT3 Females vs. HDID-2:ZT15 Females	****	<0.0001
**Selected focus: within ZT, line difference for each sex**		
HS/Npt:ZT3 Females vs. HDID-1:ZT3 Females	****	<0.0001
HS/Npt:ZT3 Females vs. HDID-2:ZT3 Females	****	<0.0001
HS/Npt:ZT15 Males vs. HDID-1:ZT15 Males	****	<0.0001
HS/Npt:ZT15 Males vs. HDID-2:ZT15 Males	****	<0.0001
HS/Npt:ZT15 Females vs. HDID-2:ZT15 Females	****	<0.0001
HDID-1:ZT15 Females vs. HDID-2:ZT15 Females	****	<0.0001

Table S2. Ethanol consumption and achieved blood alcohol levels at multiple time points during a 4 h Drinking in the Dark assay in Hs/Npt, HDID-1, and HDID-2 mice: Tukey's post-hoc results for BAL analysis. (supplement to Figure 2b).

Time	Comparisons	Summary	Adjusted p Value
20 min	HDID-1 vs. HDID-2	ns	0.4768
	HDID-1 vs. HS/Npt	ns	0.7794
	HDID-2 vs. HS/Npt	ns	0.8762
40 min	HDID-1 vs. HDID-2	ns	0.6348
	HDID-1 vs. HS/Npt	ns	0.6893
	HDID-2 vs. HS/Npt	ns	0.9959
60 min	HDID-1 vs. HDID-2	ns	0.6686
	HDID-1 vs. HS/Npt	ns	0.4999
	HDID-2 vs. HS/Npt	ns	0.9609
80 min	HDID-1 vs. HDID-2	***	0.0006
	HDID-1 vs. HS/Npt	****	<0.0001
	HDID-2 vs. HS/Npt	ns	0.752
100 min	HDID-1 vs. HDID-2	ns	0.4788
	HDID-1 vs. HS/Npt	ns	0.5499
	HDID-2 vs. HS/Npt	ns	0.0732
120 min	HDID-1 vs. HDID-2	ns	0.8817
	HDID-1 vs. HS/Npt	****	<0.0001
	HDID-2 vs. HS/Npt	****	<0.0001
140 min	HDID-1 vs. HDID-2	**	0.007
	HDID-1 vs. HS/Npt	****	<0.0001
	HDID-2 vs. HS/Npt	*	0.01
160 min	HDID-1 vs. HDID-2	ns	0.2247
	HDID-1 vs. HS/Npt	****	<0.0001
	HDID-2 vs. HS/Npt	****	<0.0001
180 min	HDID-1 vs. HDID-2	****	<0.0001
	HDID-1 vs. HS/Npt	ns	0.8936
	HDID-2 vs. HS/Npt	****	<0.0001
200 min	HDID-1 vs. HDID-2	ns	0.1585
	HDID-1 vs. HS/Npt	****	<0.0001
	HDID-2 vs. HS/Npt	**	0.0012
220 min	HDID-1 vs. HDID-2	****	<0.0001
	HDID-1 vs. HS/Npt	****	<0.0001
	HDID-2 vs. HS/Npt	****	<0.0001
240 min	HDID-1 vs. HDID-2	**	0.0014
	HDID-1 vs. HS/Npt	****	<0.0001
	HDID-2 vs. HS/Npt	****	<0.0001

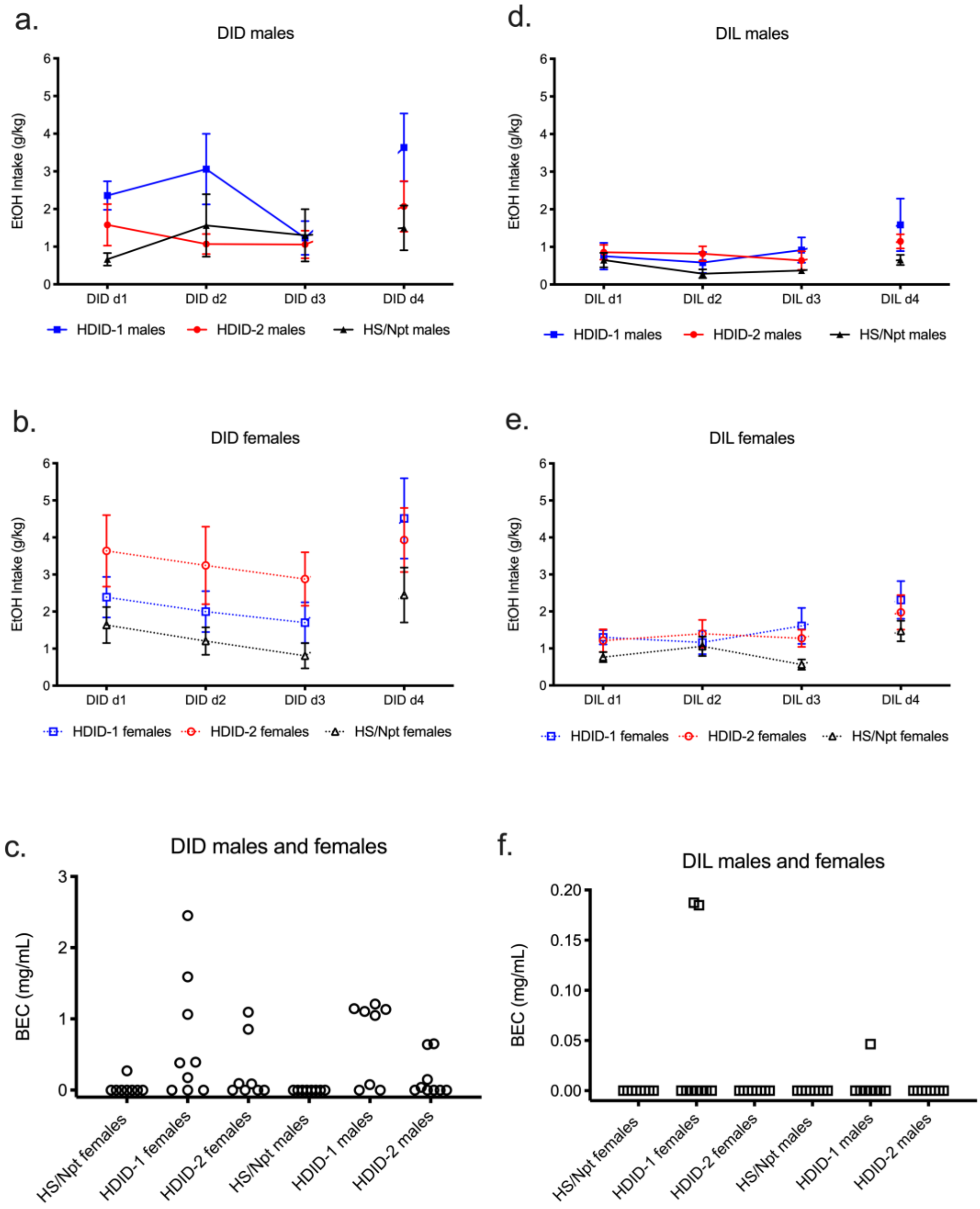

Figure S1. Ethanol consumption and achieved blood alcohol levels for Hs/Npt, HDID-1, and HDID-2 mice in Drinking in the Dark and Drinking in the Light assays: males and females shown separately. Male and female Hs/Npt, HDID-1 and HDID-2 mice were tested over four days for ethanol consumption and resulting BALs in both the dark and the light. Ethanol intakes for days 1-3, 2hr each day,and 4 hr on day 4 are shown for drinking in the dark (**a**. male intake, **b**. female intake, and **c**. BECs) and drinking in the light (**d**. male intake, **e**. female intake, and **f**. BECs). Results are presented in the main manuscript.

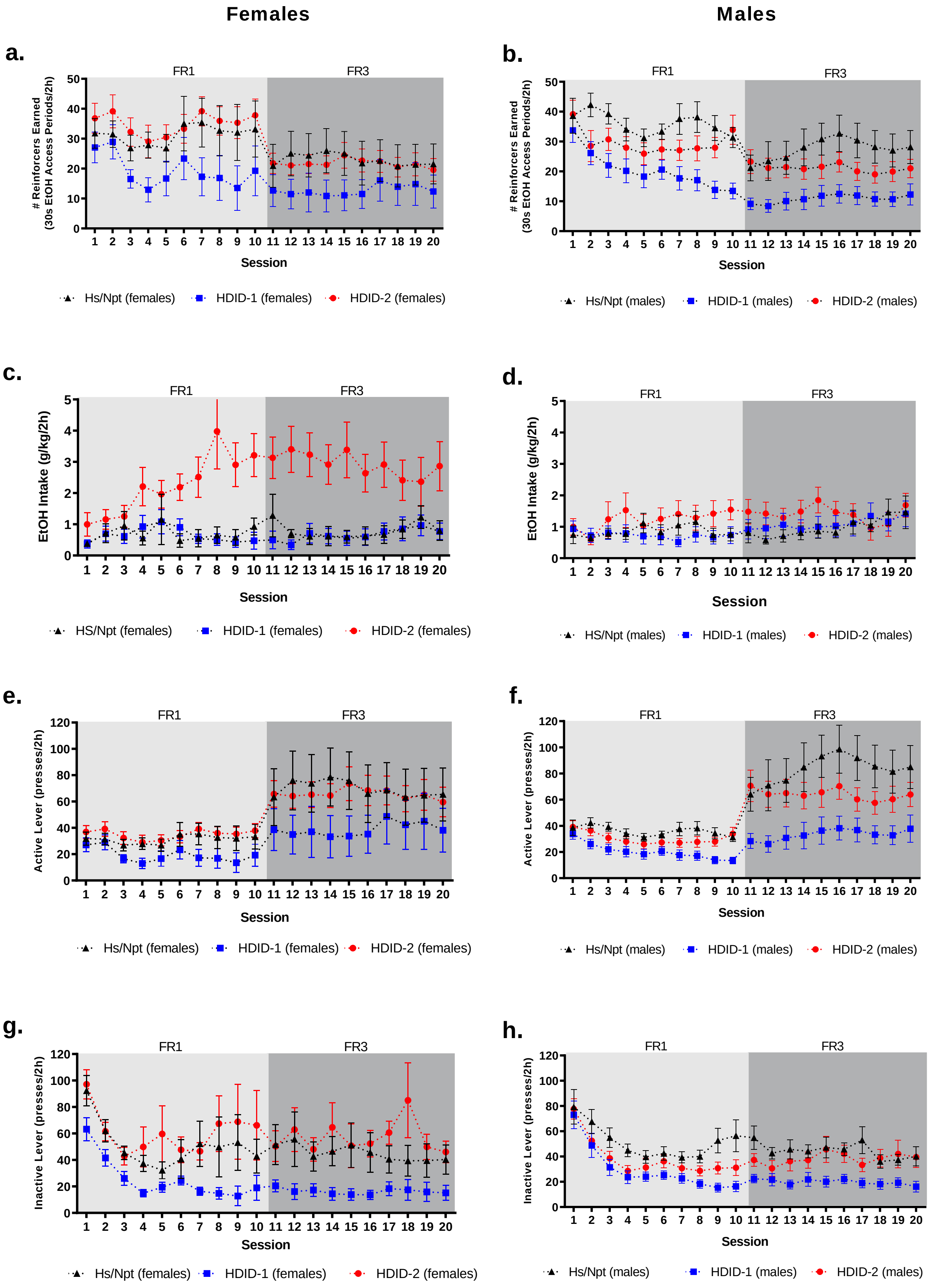

Figure S2. Differential operant ethanol self-administration behaviors observed in Hs/Npt, HDID-1, and HDID-2 mice: data for all sessions. The self-administration operant data for each sex (separately) and genotype, showing the average # of access periods (**a**, **b**), EtOH intake (**c**, **d**), active lever presses (**e**, **f**) and inactive lever presses (**g**, **h**) per session. FR1 testing is on the left of each chart in light grey, FR3 testing on the right in darker grey.

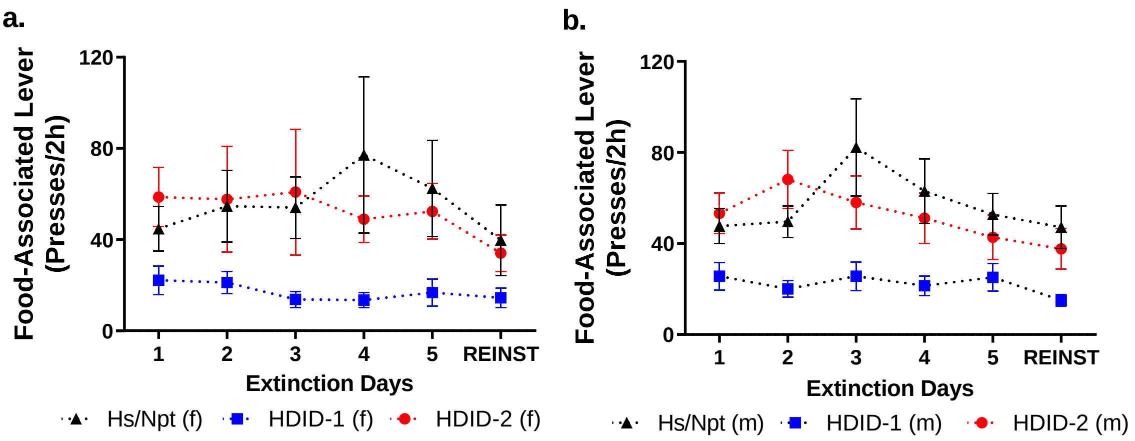

Figure S3. Hs/Npt, HDID-1, and HDID-2 mice behaviors under extinction and cue-induced reinstatement conditions: previously food-associated lever presses (this lever was inactive during ethanol sessions). Mice underwent five sessions of extinction and one session of reinstatement after completing FR3. Data for each genotype's pressing of the previously food-associated lever (inactive during ethanol FR1 and FR3 testing) for females (**a**) and males (**b**) is shown here. For graphs on pressing of the active (formerly EtOH-associated) lever, see **Figure 7**.

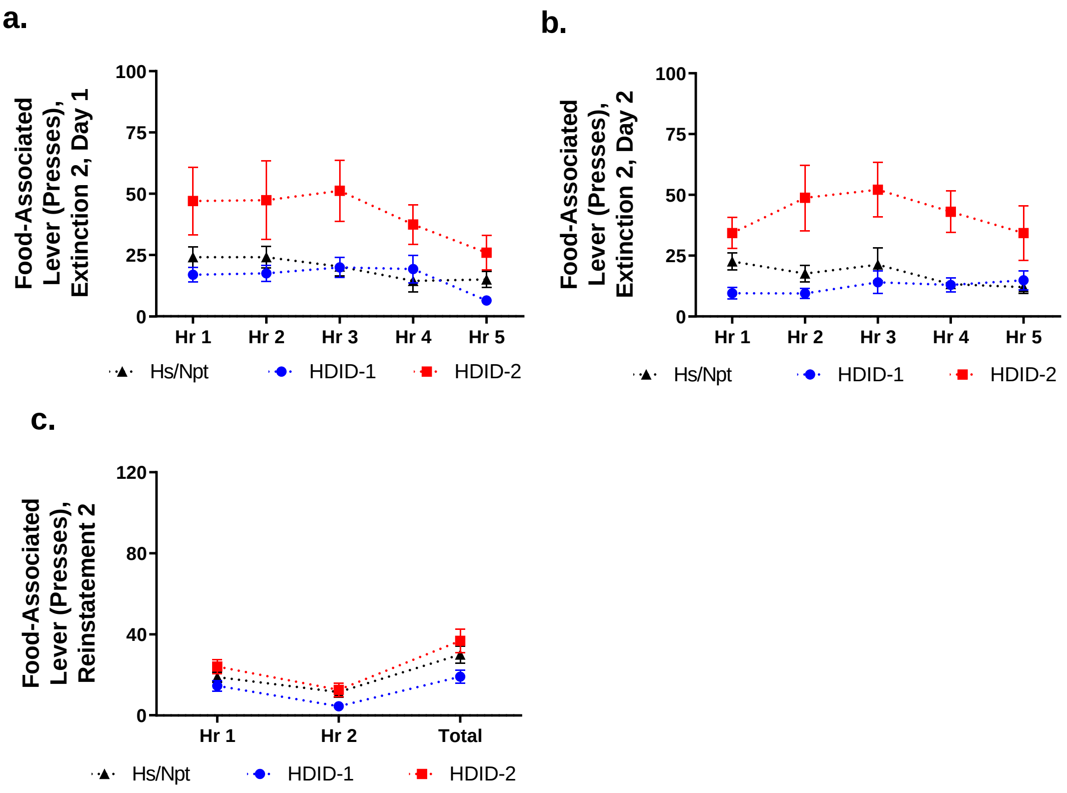

Figure S4. Hs/Npt, HDID-1, and HDID-2 mice behaviors under additional extinction and cue-induced reinstatement conditions: previously food-associated lever presses (this lever was inactive during ethanol sessions). One cohort of mice underwent a second set of extinction (Extinction 2: 2 sessions, 5 hours each) and reinstatement (Reinstatement 2: 1 session, 2 hours, FR3, water only in sipper tubes). Data shown here is the hourly breakdown of the formerly food-associated lever (which has been inactive since the start of ethanol FR1) presses for Extinction 2, Day 1 (**a**); Extinction 2, Day 2 (**b**); and Reinstatement 2 (**c**). Sexes are collapsed across genotypes. For graphs on the formerly EtOH-associated lever presses, see **Figure 8**.
